# Fibrolytic capacity of the gut microbiome in captive African elephants (*Loxodonta africana*): a pilot study

**DOI:** 10.3389/fvets.2026.1933152

**Published:** 2026-09-30

**Authors:** Barbara U. Metzler-Zebeli, Folko Balfanz, Fritz Vollrath

**Affiliations:** 1 Unit Nutritional Physiology and Functional Plant Compounds, University of Veterinary Medicine Vienna, Vienna, Austria; 2 Vienna Zoo, Tiergarten Schönbrunn, Vienna, Austria; 3 Department of Biology, University of Oxford, Oxford, United Kingdom; 4 Save the Elephants, Marula Manor, Nairobi, Kenya

**Keywords:** African elephant, carbohydrate-active enzymes, fermentation, gut microbiota, plant polysaccharide breakdown

## Abstract

African elephants (*Loxodonta africana*) depend on the fibrolytic capacity of their gut microbiome (GMB) to efficiently utilize fibrous plant material. However, knowledge is missing on the temporal stability and functional dynamics (especially fibrolytic capacity) of their GMB. Likewise, information on the associated volatile fatty acids (VFA) production in the elephant’s hindgut is scarce. In this pilot study, we collected feces from five captive African elephants on two occasions separated by 12 months to explore the GMB composition and its changes over time. We characterized the GMB’s fibrolytic potential based on total microbial numbers and metagenome composition, including carbohydrate-active enzymes (CAZy), and fecal VFA, at individual and group level. Elephants received a roughage-based diet, which contained more carrots at the second than at the first sampling. Bacteria, specifically fibrolytic *Prevotella*, *Bacteroides*, *Ruminococcus* and *Fibrobacter* dominated the GMB, while archaea, fungi and protozoa were less present. *Treponema* was highly abundant, which might be an adaptation to maximize energy gain from cellulose-rich food. The elephants showed individual shifts in GMB composition from the first to second sampling. The shift in functional gene composition from the first to the second sampling was similar among elephants. It showed distinct clustering of CAZy families between fecal collections and discriminated features related to breakdown of carrot pectin for the second sampling. Sparse partial least squares-discriminant analysis identified (hemi-)cellulolytic genera within *Prevotellaceae* as discriminant taxa, which positively associated with CAZy families but negatively with VFA. At group level, fecal VFA concentrations were similar among elephants. However, opposite shifts from the first to the second sampling were detected among elephants at individual level. It may be speculated whether feeding decisions of three related females might explain the increase of VFA in their feces at the second compared to the first sampling, whereas VFA were lower in feces of the non-related female at the second sampling. We assert that this pilot study on GMB taxonomy and carbohydrate breakdown in captive African elephants may be helpful for feeding management at zoological gardens.

## 1 Introduction

The gut microbiome (GMB) provides physiological benefits and costs to its host and thus plays a crucial role in its well-being (1). For instance, the gut microbes contribute digestive enzymes important for the breakdown of plant materials, trigger immune responses and influence hormonal and neurological processes. Moreover, the commensal GMB helps protecting its host against pathogenic invasion (2). Simultaneously, the physiology of the host and the environmental conditions to which the host is exposed can modulate the GMB composition (3). Based on this knowledge, research interest into the influence of the GMB on health and disease of wild and captive Asian (*Elephas*) and African (*Loxodonta*) elephants has increased in recent years. An improved understanding of their eco-physiology, nutritional and environmental needs is useful information for their conservation management (4–8). In particular, it is important to learn more about the mechanisms that drive variation in the GMB and its temporal stability over time as well as to deviate potential consequences for elephant survival, health and well-being at the individual and group level (4, 8). It can be helpful to study the elephant’s GMB and its functional capacities in zoological facilities (9), where both environment and food can be better controlled. However, studying the GMB in captive elephants has its limitations to serve as model for natural microbial ecology; especially when the elephants are kept in other regions than their natural habitat. For instance, factors like the composition of the captive diets and foraging behavior, reduced environmental microbial exposure, altered social interactions and transfer of animals between zoological facilities, veterinary interventions, and climate can all alter the GMB composition compared to their wild counterparts (9, 10). Nevertheless, elephants in captivity are likely to benefit from a better understanding of individual differences in their GMB composition and its capacity to digest food, allowing for a greater attention to individual dietary needs to promote (gut) health, longevity and reproductive rate (9). Changing the type and abundance of available food is a major extrinsic factor for GMB composition and metabolic functions (7, 11). A consistent diet, as often fed in zoological facilities, can be used to simulate a reduced food diversity. On the one hand, such a diet can result in the enrichment of certain microbial taxa and metabolic capacities (i.e., lignocellulolytic capacities) (12) and may even influence the host’s preferences for these foods (13). On the other hand, limited dietary diversity may lead to long-term microbial community shifts (7), which, in turn, may lead to loss of important metabolic functions and a decreased ability to adapt to dietary changes (7, 12, 14).

As mega herbivores, elephants rely on the efficacy of their GMB to extract energy in the form of volatile fatty acids (VFA) from plant polysaccharides, which the elephant then absorbs. Simultaneously, elephants are bulk eaters with a relatively short retention time of food in the gut, which limits the time for the microbes to ferment their cellulose-rich diet and to produce VFA (15, 16). This requires a high fermentation and absorptive efficiency to meet the energy demand of the host. Metagenome analysis revealed a high diversity of cellulose-degrading bacteria and glycoside hydrolase (GH) family enzymes in Asian elephants (3, 12), which were increased in the GMB of elephants living in captivity compared to their wild counterparts (12). While substantial knowledge has been gained about the taxonomic composition (5, 7, 11, 12, 17), information about the fibrolytic capacity of the GMB and the associated VFA production is still limited, especially in African elephants (17). Aside from the taxonomic and functional composition, the dynamics in the total microbial load, including bacteria, fungi, protozoa and archaea, is an important component in the gut microbe–host interplay, which have been hardly described for elephants.

In the present pilot study, we collected feces from captive African elephants at two occasions separated by 12 months to characterize their fibrolytic potential based on fecal VFA, total microbial numbers and metagenome composition, including carbohydrate-active enzymes (CAZy), at individual and group level. The five elephants (four females and one male) were all offered the same roughage-based diet, which contained carrots as a staple at the second but only as ‘treats’ at the first fecal sampling. We hypothesized that the GMB of the five zoo elephants would be adapted to lignocellulose metabolism and comprise a diverse cellulolytic community. We further hypothesized that a higher availability of readily fermentable dietary carbohydrates in the form of carrots would lead to microbial and functional shifts related to hemicellulose breakdown, but that the individual differences in the gut metagenome communities among elephants would persist.

## 2 Materials and methods

### 2.1 Housing, feeding and feces collection

Fecal samples were collected in Vienna Zoo (Tiergarten Schönbrunn, Vienna, Austria) from five adult African elephants (*Loxodonta africana*). The five elephants comprised the unrelated bull (E1, in Vienna Zoo since 2023), the unrelated female E2 (in Vienna Zoo since 2009) and three related females, i.e., E4 and her 2 daughters E3 and E5 (Supplementary Table 1). Female E4 is the matriarch of the small elephant group and has been in Vienna since 1998 where she gave birth to her daughters in 2013 (E3) and 2003 (E5). The elephants were group-housed with indoor and outdoor areas with additional single pens for each elephant. On the days prior to the fecal collections, all elephants were offered the same diet, but the dietary composition changed from the first to the second sampling. In December 2023, their forage consisted of reed hay and meadow hay (ratio 70:30 w/w), with additional bamboo and beech branches with leaves (ratio 90:10 w/w). In December 2024, the elephants foraged on reed hay, meadow hay, bamboo and beech branches (similar to 2023) and carrots which were provided between 10 and 11 a.m. daily. In both years, vegetables (e.g., carrots and onions) and fruits (e.g., apples) were provided as marginal ‘treats’ (training rewards).

Fecal samples were collected on two occasions separated by 12 months in December 2023 and 2024. Only fresh feces, which had to be moist and warm, laying in the indoor area were collected at 13:00 h on both collection days. To be able to access the feces, the elephants had to be deployed from the indoor area to the outdoor area or to their respective pen. Video-tracking was used to ensure that all elephants had defecated as well as to link elephant and dung. Accordingly, fresh fecal samples were collected within 1 h of defecation (with a maximum of tolerance of 2 h after defecation if no subsequent defecation occurred). Fecal matter (approximately 500 g) from the inner part of the fecal balls were aseptically collected to reduce environmental contamination from the floor, bedding, and exposure to air (oxygen). The feces were flattened in sterile freezing bags for easier subsampling and cutting when frozen. Immediately after the collection, the samples were transported on ice to the laboratory at University of Veterinary Medicine Vienna, where they were stored at −20 °C for DNA extraction and VFA analysis. The frozen flattened feces were broken in pieces to aliquot them for the two analyses. All samples from both collections were treated similarly and processed within two months of collection.

### 2.2 DNA extraction

Genomic DNA was extracted from feces in one run after each of the two samplings. The DNA was extracted using a commercial DNA extraction kit with modifications to the manufacturer’s protocol (DNeasy PowerSoil Pro Kit, Qiagen, Hilden, Germany). The modifications were established prior to DNA extraction of the current samples, partly adapted from the DNA extraction protocol for fibrous ruminal content (18). Due to the high content of structural fiber in elephant’s feces, about 50 g from different parts of the frozen collected fecal matter was manually cut to 2–5 mm. It was paid attention that feces did not thaw during this process. Between analytical steps, the tubes containing the samples were kept on ice. Of the finely chopped and mixed feces, 500 mg were used for DNA extraction, which were placed into a bead beating tube containing 1,000 μL of C1 buffer. Additional heating and enzymatic treatment were employed to facilitate the lysis of microbial cells attached to the fibrous material in feces (18). Sample tubes were incubated at 95 °C for 5 min. Samples were centrifuged, and the supernatant was collected in a fresh tube and placed on ice. The pellet was mixed with 100 μL lysozyme (100 mg/mL; Sigma-Aldrich Inc., St. Louis, MO, USA) and 10 μL mutanolysin (2.5 U/mL; Sigma-Aldrich Inc.) and incubated at 37 °C for 30 min. Thereafter, 21.3 μL of proteinase K (18.8 mg/mL, Sigma-Aldrich Inc.) was added to the pellet and incubated again at 37 °C for 60 min. Pellets and supernatant were combined and placed in a SpeedMill (Analytik Jena GmbH + Co KG, Jena Germany). Samples were homogenized using a three-interval bead beating program of 1-min homogenizations and 1-min rest. The supernatant was collected and the DNA extraction was continued according to the kit manual. The total DNA was eluted using 100 μL C6 buffer. Total DNA quantity was determined by means of the Qubit Fluorometer 4.0 (Life Technologies Corporation, Carlsbad, USA) and the Qubit dsDNA HS Assay Kit (Thermo Fisher Scientific, Vienna, Austria) according to the manufacturer’s instructions. All DNA extracts were stored at −20 °C. Aliquots were used for quantification of total microbial abundances and high-throughput sequencing.

### 2.3 Quantitative PCR

Absolute quantification of total bacteria, archaea, fungi and protozoa in fecal DNA extracts was performed on a qTOWER real-time PCR system (Analytik Jena GmbH, Jena Germany) using previously reported primer sets (Supplementary Table 2) and amplification conditions (19). Each reaction (total volume 10 μL) consisted of 0.5 ng DNA, 2.5 μL innuMIX qPCR DSGreen Standard (Analytik Jena GmbH), 300 nM each of forward and reverse primers, and DEPC-treated water (G-Biosciences, St. Louis, MO, USA) in a 96-well plate. After an initial denaturation at 95 °C for 2 min, 40 cycles of 95 °C for 30 s and primer annealing and elongation at 60 °C for 60 s were performed. Subsequently, melting curve analysis was performed with an increment of 0.1 °C/s from 60 to 95 °C, with fluorescence measurement at 0.1 °C intervals. Standard curves were prepared by making serial dilutions (10^10^ to 10^3^ molecules/μl) of the purified and quantified PCR products generated by use of pooled DNA from *Loxodonta* feces. Standards were run on the same plate in triplicate, while samples and negative controls without template DNA were included in duplicate. Amplification efficiency (E = 10^–1/slope^) and coefficient of determination can be found in Supplementary Table 2. The final gene copy numbers per gram of feces were calculated using the following equation: (QM × C × DV)/(S × V), where QM is the quantitative mean of the copy number, C was the DNA concentration of each sample, DV is the dilution volume of extracted DNA, S is the DNA amount (ng) subjected to analysis, and V is the weight of the sample (g) subjected to DNA extraction.

### 2.4 Shotgun metagenomics

Shotgun metagenomics sequencing was performed at an external laboratory (Novogene UK, Cambrigde, UK) including library preparation (NEBNext DNA Library Prep Kit, Illumina, San Diego, CA, USA). After quality check of the libraries, different libraries were pooled according to their effective concentrations and target data output requirements and then subjected to paired-end 150 sequencing on an Illumina NovaSeq 6,000 sequencer. Adapter sequences were trimmed using fastp (https://github.com/OpenGene/fastp; version 0.23.1). Reads with a mean quality score below 10 and a length less than 50 bp were removed to obtain high-quality paired-end reads. The quality filtered sequences were spliced and assembled using MEGAHIT (version 1.2.9) (20, 21). Open reading frame (ORF) prediction for scaftigs (≥ 500 bp) of each sample were performed using MetaGeneMark (http://topaz.gatech.edu/GeneMark/; version 2.1) (22). Genes with < 100 bp were filtered out (23, 24). The CD-HIT software (http://www.bioinformatics.org/cd-hit/; version 4.5.8) was used for the dereplication of the ORF results and to obtain the non-redundant initial gene catalogue (23). Clean data of each sample were aligned to the initial gene catalogue by using Bowtie2 (http://bowtie-bio.sourceforge.net/bowtie2/index.shtml; version 2.5.4) to calculate the number of reads of the genes on each sample alignment (25, 26). Genes with reads ≤ 2 in each sample were filtered out to obtain the final gene catalogue (Unigenes) for subsequent analysis (23). The Unigene sequences of the non-redundant gene sets were aligned to the Micro_NR database (version 2024.03), which includes sequences from bacteria, fungi, archaea and viruses (BLASTP alignment parameters: e-value of 1e-5) using DIAMOND (https://github.com/bbuchfink/diamond/; version 2.1.9) (20, 27). Next, the Unigene sequences were aligned with the functional databases with an e-value threshold set to 1e-5. Functional databases included the CAZy database (Carbohydrate-Active Enzymes Database; http://www.cazy.org/; version 2024.03) (28).

Sequence data were compositionally normalized by converting them into relative abundances by collapsing the raw sequence counts from the taxa and functional gene tables such that each sample summed to 1 and multiplied by 100 to obtain percentages. Relative gene abundance tables for each sample at each taxonomy and functional level were acquired. For taxonomy and functional level, the compositionally normalized data were used to calculate alpha-diversity indices including Shannon and Simpson using the R package vegan (version 2.7–2; October 8, 2025) (29).

### 2.5 Volatile fatty acid analysis

The concentrations of acetate, propionate, isobutyrate, butyrate, isovalerate, valerate, caproate, and heptanoate in feces were analyzed using gas chromatography (GC). All samples were prepared in one run from the originally frozen fecal pieces. One gram of finely cut feces were mixed with 200 μL of 25% ortho-phosphoric acid (4.36 mol/L), 300 μL of 4-methylvaleric acid (23.83 μmol/mL) as internal standard (Sigma-Aldrich, St. Louis, MO, USA) and 1 mL of double-distilled water. Samples were quickly processed in an ultrasonic bath, mixed thoroughly, and centrifuged (20,000 × g at 4 °C for 20 min). Volatile fatty acids were measured in the clear supernatant on the GC-2010 Plus Capillary GC (Shimadzu Corp., Kyoto, Japan) using a 30 m × 0.53 mm × 0.5 μm capillary column (Trace TR Wax, Thermo Fisher Scientific, Waltham, MA, USA) and helium as carrier gas. The injection volume was 1 μL. The GC was set up with an autosampler and injector (AOC-20s Auto Sampler; AOC-20i Auto-Injector, Shimadzu Corp., Kyoto, Japan) and a flame-ionization detector (FID-2010 Plus, Shimadzu Corp., Kyoto, Japan). The chromatographic conditions were the following: injector temperature: 200 °C, inlet pressure: 36 kPa; injection method: split (1:10); injection volume: 0.5 μL; total run time: 12.2 min; detector temperature: detector: 220 °C. The oven temperature program is shown in Supplementary Table 3. Quantification of individual VFA was performed with a single-point internal standard calibration. A standard mixture containing defined concentrations of each VFA, the same concentration of the internal standard (4-methylvaleric acid) and 25% ortho-phosphoric acid as in the samples was analyzed. For the normalization of the peak areas, the peak areas of the target VFAs were normalized against the peak area of the internal standard to compensate for injection variability and matrix effects. The relative response factor for each individual VFA was calculated using the peak area from the chromatogram, molecular weight of the respective VFA and its concentration in the GC vial. Subsequently the concentration of individual VFAs in the samples was quantified using the specific relative response factor. The reliability of compound identification and measurement was confirmed through a combination of duplicate sample preparation, replicate chromatographic injections, and regular checks of relative response factors of VFAs. The limits of detection and quantification are provided in Supplementary Table 4. The results for the VFA are presented on the basis of wet feces, as this reflects the condition in the large intestine and thus the interaction of the VFA in digesta with the gut epithelium.

### 2.6 Statistical and multivariate analysis

The compositionally normalized relative abundance data were used for statistical and multivariate analysis. First, the analysis of similarity (ANOSIM) with the ‘anosim’ function in R was conducted for both the taxonomic community and the functional composition. Next, PERMANOVA (adonis, permutations = 999) using the relative abundance data was used to assess inter-sampling and inter-animal differences in beta-diversity based on Bray-Curtis distance. In addition, two-dimensional non-metric multidimensional scaling (NMDS) analysis was performed using the ‘metaMDS’ function in R to assess differences in beta-diversity between years and individual animals based on Bray-Curtis distance. Both analyses describe community dissimilarities, whereby the advantage of the ANOSIM is that the R statistic values allow direct comparison of groups with other studies. The disadvantage of ANOSIM is that there is a potential risk that it confounds the differences between groups and dispersion within groups. Therefore, we also conducted the PERMANOVA based on pseudo-*F* values to detect changes in community structure for the present microbial community (30). The ggplot2 package was used to visualize the clustering of taxonomic and gene communities. Hierachical clustering was performed using hclust and the corresponding dendogram was created using the dendextend package (version 1.19.1), which was visualized using the heatmap.2 function in gplots (version 3.3.0).

In order to deepen the understanding about potential relationships between taxonomic (genera) and functional (CAZy level 2) composition and VFA concentrations in feces, multigroup supervised DIABLO N-integration was performed to identify the key features and their relations among each other by means of the package ‘mixOmics’ (version 6.3.2) (31). Supervised multigroup sparse partial least squares-discriminant analysis (sPLS-DA) was used to integrate datasets of relative abundances of microbial genera, CAZy level 2 genes, GH genes and VFA concentrations to classify and select key features from each dataset. Two sPLS-DA were performed: the first including all CAZy level 2 genes; the second with only GH genes. Tuning of sPLS-DA parameters was performed using the ‘tune’ function to identify the most influential genera, CAZy level 2 genes and VFA with the lowest possible error rate. The ‘tune’ function returns a set of ‘keepX’ values for each dataset that shows the best predictive performance for all the components in the model, while simultaneously performing repeated leave-one-out cross-validation using the function ‘nrepeat’ (31). In the first sPLS-DA, we tuned the number of retained variables to one fourth of parameters for the microbial (> 0.08% relative abundance) and CAZy level 2 gene datasets (> 0.03% relative abundance), while keeping most VFA for components 1 and 2, respectively. The second tuning retained variables to 5% of parameters for microbial and GH genes, and four VFA. The sPLS-DA results were visualized in sample projection plots, loading plots and circos plots for the most discriminant variables for each subset of data and component 1. Circos plots present the strongest positive and negative Pearson’s correlation (|r| > 0.8) between the most discriminant genera, CAZy level 2 or GH genes, and VFA concentrations.

Differences in fecal VFA concentrations, total microbial gene copy numbers, alpha-diversity, as well as relative abundances of the 20 most abundant taxa (genus level) and CAZy level 2 genes between sampling time points were tested by means of linear models (ANOVA) and repeated measures over time with ‘fecal sampling’ as fixed effect using the mixed procedure in SAS (SAS Stat Inc., version 9.4; Cary, NC, United States) after analyzing for normality using the Shapiro–Wilk test. The random effect was the individual elephant. If residuals were not normally distributed, the Boxcox method and the Transreg procedure were used to transform data before subjecting the data to ANOVA. Degrees of freedom were approximated using the Kenward-Roger method. Differences among least squares means were computed using the pdiff statement and the Bonferroni correction was applied to adjust raw *p*-values. Differences were considered significant if *p* ≤ 0.05 and as trend if 0.05 < *p* ≤ 0.10.

## 3 Results

### 3.1 Volatile fatty acids and total microbial abundances

Total VFA concentrations ranged from 67 to 102 mmol/kg feces and were highest in the feces of the oldest female E4 (matriarch) compared to the other elephants (Figure 1A). At group level, the ANOVA indicated similar total and single VFA concentrations across the two sampling time points (*p* > 0.05; Figures 1A–F; Supplementary Table 5). However, when comparing the individual elephants, VFA concentrations showed temporal differences from the first to second fecal collection (Figures 1A–G). Responses diverged in that the concentration of VFA were similar (E1, male), lower (E2, female) or higher (E3 to E5, females) on the second sampling compared to 12 months earlier. At individual level, females E3, E4 and E5 had 19 to 22%-higher acetate, propionate and butyrate concentrations in feces on the second compared to the first sampling. The feces of the four females contained a higher proportion of branched-chain fatty acids compared to the male (Supplementary Table 6). Bacteria were abundant at 10 log_10_ gene copies/g feces and the ANOVA at group level showed that their number remained stable over time (Figure 2A). Also at group level, archaeal and protozoal gene copies increased by 0.7 log units at the second fecal sampling compared to 12 months earlier (*p* < 0.05; Figures 2B,C), whereas fungal gene copies decreased by 0.7 log units (*p* = 0.029) during the same period (Figure 2D). At individual level, the changes were generally similarly directed among the individual elephants (Figures 2A–D), with E3 and E4 having higher archaeal gene copy numbers compared to the other three elephants. Of note, protozoal gene copies increased most in E1 and E5 as they raised by 1.1 and 1.3 log units, respectively, between the two sampling time points.

**Figure 1:**
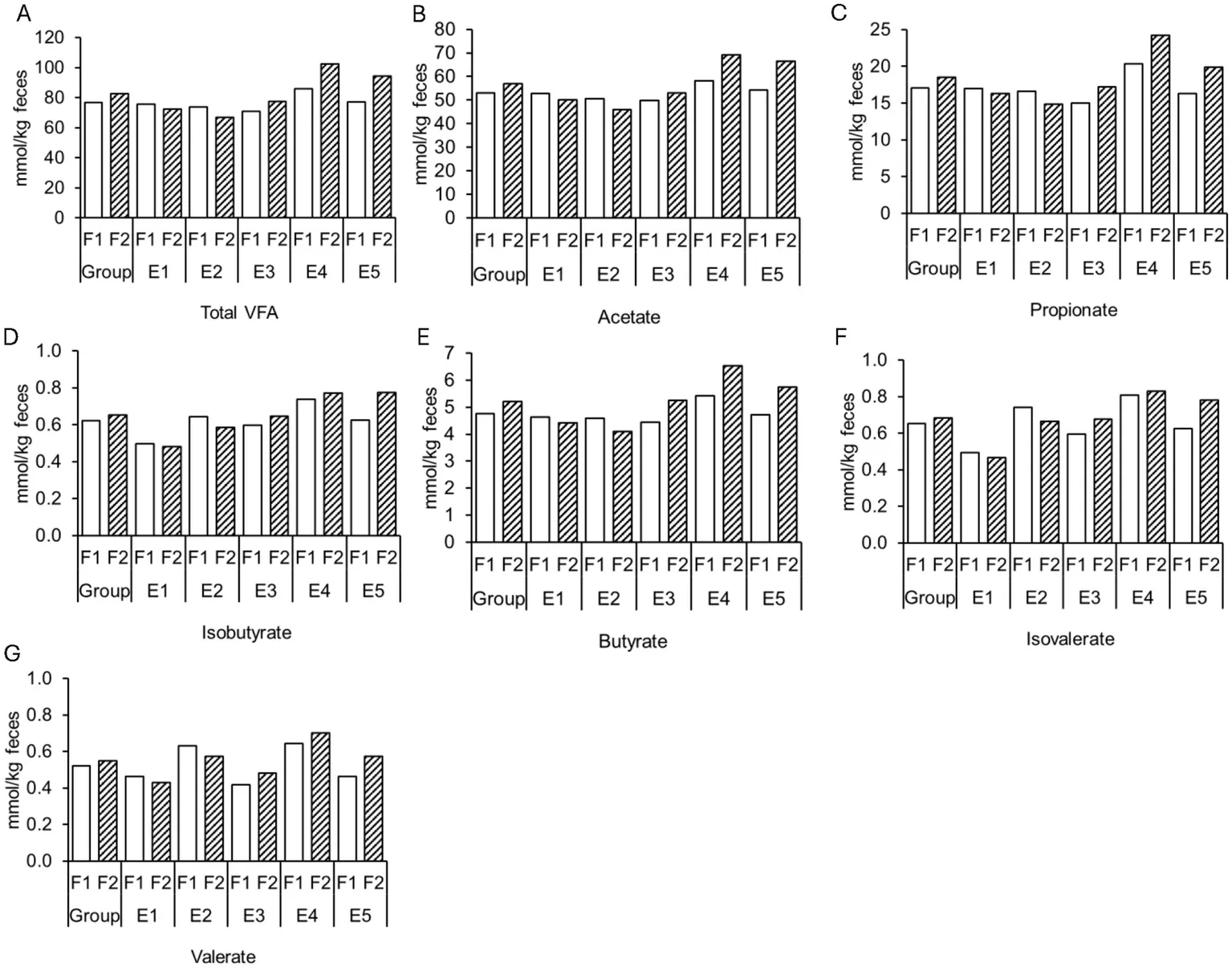
Comparison of total and single volatile fatty acid (VFA) concentrations at group and individual level **(A–G)** between fecal collections. F1, fecal collection in 2023; F2, fecal collection in 2024. E1, male; E2–E5, females. Group values for F1 and F2 are least-squares means and were not different between F1 and F2. The standard error of the mean and exact *P* values can be found in Supplementary Table 5.

**Figure 2:**
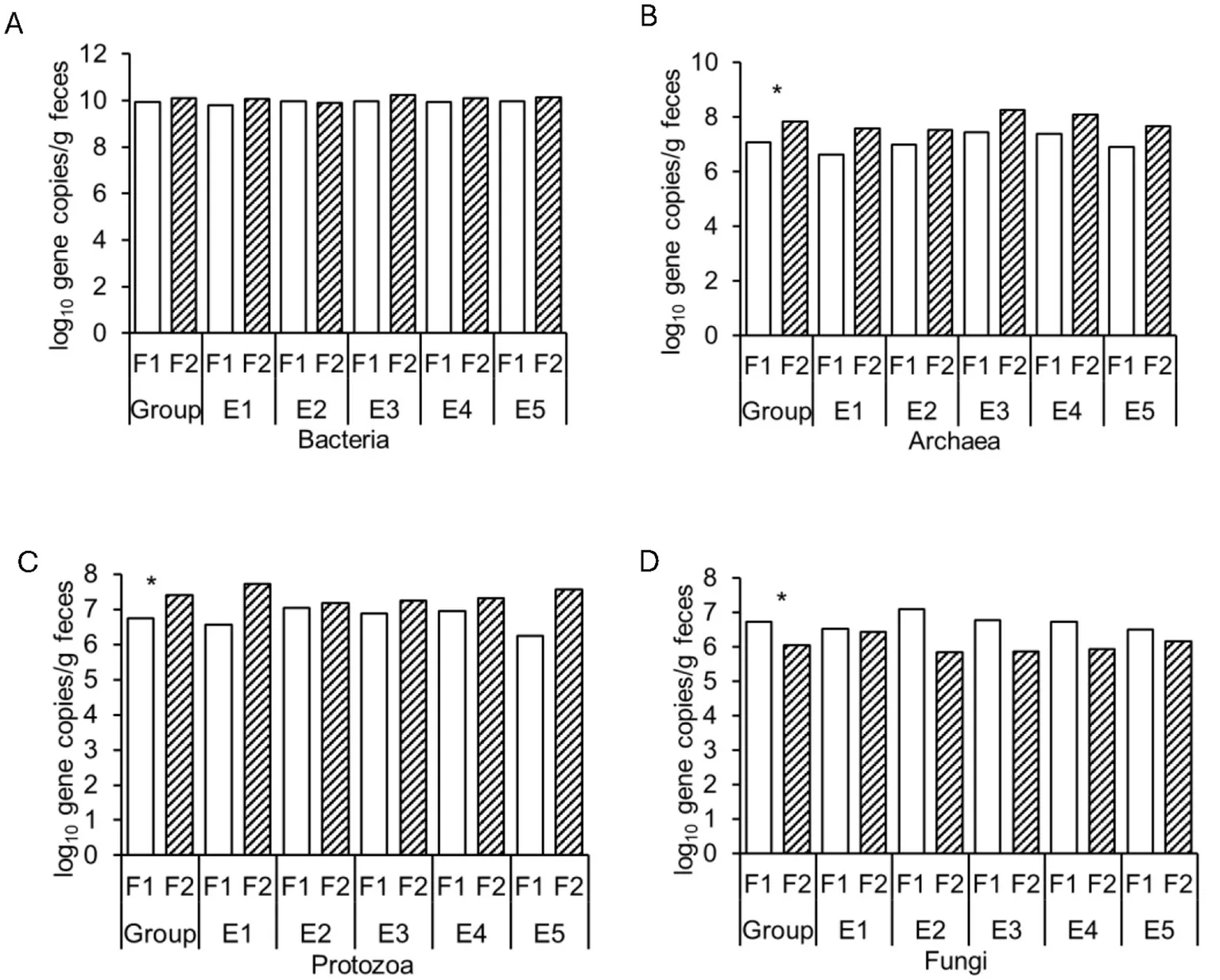
Comparison of the total microbial gene copies at group and individual level between fecal collections. F1, fecal collection in 2023; F2, fecal collection in 2024. E1, male; E2–E5, females. Group values for F1 and F2 are least-squares means. **(A)** Bacteria: Group, standard error of the mean (*SEM*) = 0.04 log_10_ gene copies/g feces, *p* = 0.049; **(B)** Archaea: Group, *SEM* = 0.15 log_10_ gene copies/g feces, *p* < 0.001; **(C)** Protozoa: Group, *SEM* = 0.11 log_10_ gene copies/g feces, *p* = 0.029; and **(D)** Fungi: *SEM* = 0.13 log_10_ gene copies/g feces, *p* = 0.043. *indicates differences (*p* < 0.05) between F1 and F2.

### 3.2 Metagenome assembly

We obtained a total of 41.5 Gbase data for the ten fecal samples. After quality filtering, a total of 41.31 Gbase of high-quality microbial sequences remained. The average total length, average length and N50 length were 251,320,332, 1,387, and 1,740 bp, respectively. The average number of scaftigs was 181,599 per sample. Gene prediction and abundance analysis showed a total number of 2,314,470 non-redundant genes and total and average length of 1,230 Mbp and 699 bp genes, respectively. Predicted genes were annotated in the CAZy database.

### 3.3 Taxonomic composition at group and individual level

In total, we detected 35 phyla above 0.01% relative abundance [30 bacterial, 2 archaeal, 1 viral and 2 Eukaryota phyla; Supplementary Table 7]. A total of 267 microbial genera were at a relative abundance >0.01% of all reads: *Treponema* (5.0%), *Prevotella* (4.0%) and *Bacteroides* (4.0%) were the dominating genera followed by *Fibrobacter* (1.8%), *Ruminococcus* (1.7%), *Clostridium* (1.4%), *Alistipes* (1.2%) and *Methanocorpusculum* (0.97%) as the major archaeal taxon (Figure 3A). From the most abundant genera, ANOVA indicated that, at group level, *Bacteroides* was less abundant, whereas the relative abundance of *Clostridium* increased at the second sampling compared to 12 months earlier (*p* < 0.05; Figure 3A). At individual level, substantial differences were found among elephants for fibrolytic genera including *Treponema*, *Ruminococcus* and *Fibrobacter*. Accordingly, *Treponema* was present ranging from 3.3 to 8.2% and 2.6 to 6.7% in the GMB of the five elephants at the first and second fecal sampling, respectively. Female E2 had an about 50% higher relative abundance of *Ruminococcus* at the second fecal sampling compared to the year before, whereas females E3 and E4 had an opposite shift in relative abundance of *Ruminococcus*. The relative abundance of *Fibrobacter* also fluctuated among elephants at individual level, being highest in E2 at the first fecal sampling and in E3 and E4 at the second fecal sampling.

**Figure 3:**
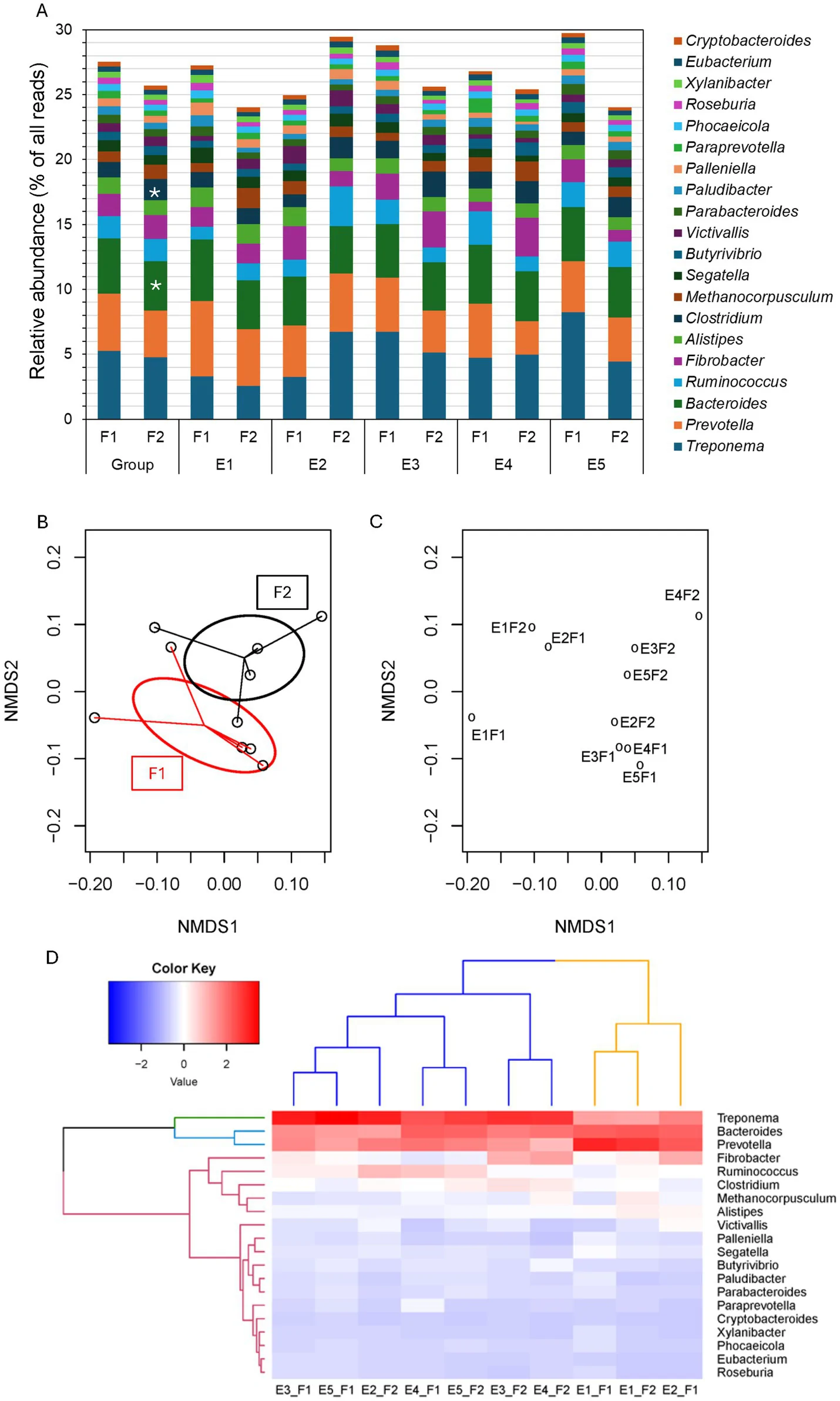
Comparison of the relative abundances of the 20 most abundant microbial genera at group and individual level between fecal collections **(A)**. F1, fecal collection in 2023; F2, fecal collection in 2024. E1, male; E2-E5, females. Group values for F1 and F2 are least-squares means; * *P* < 0.05 at group level. Non-metric multidimensional scaling plots of fecal microbiota from elephants between fecal collections (**B**; stress = 0.0404), fecal microbiota with assignment of the individual elephant **(C)**. Heatmap showing hierarchical clustering of bacterial genera across samples **(D)**. Relative abundance values were Z-score normalized and are represented on a color scale ranging from − 2 (blue, low abundance) to + 2 (red, high abundance). Rows represent bacterial genera. Columns represent fecal samples from individual elephants (E1-E5) and fecal collections (F1 and F2). Both bacterial genera and samples were clustered using hierarchical clustering to visualize similarities in community composition.

At group level, alpha-diversity, including Shannon and Simpson determined at genus level, was similar among sampling time points and individual elephants (Supplementary Table 8). According to the results for NMDS (Bray-Curtis distance) the GMB communities between fecal samplings slightly overlapped (Figure 3B). The ANOSIM analysis at the genus level supported this observation by indicating that the within group variation in GMB was higher than the variation over sampling time points (*R* = 0.03, *p* = 0.40). The ANOSIM further indicated that intra-animal variation in GMB communities between sampling time points was greater than inter-animal variation (*R* = 0.22, *p* = 0.16). Visualization of the beta-diversity of the GMB communities (Bray-Curtis distance) by identifying the individual elephants between the two time points supported these observations (Figure 3C). The results show distinct changes along the NMDS1 and NMDS2 axis for E1 and E2, whereas the GMB communities of E3, E4 and E5 mainly changed along the NMDS2 axis, with the largest change for E4. Hierarchical clustering of fecal samples further supported inter- and intra-individual differences in taxa composition from the first to the second fecal sampling (Figure 3D).

### 3.4 Functional gene comparison at group and individual level

CAZy gene families amounted to about 6.3% of all functional genes. At CAZy level 1, glycoside hydrolases (GH) genes dominated with about 2.9%, followed by glycosyl transferases (GT, 2.0%), carbohydrate-binding modules (CBM, 0.70%), carbohydrate esterases (CE, 0.33%), polysaccharide lyases (PL, 0.31%) and auxiliary activities (AA, 0.03%; Supplementary Table 9). At group level, two of the six functional categories of CAZy level 1 genes showed shifts in relative abundances from the first to the second fecal sampling. The relative GT abundance increased by 20.4% on the second sampling compared to 12 months earlier (*p* = 0.002), whereas the relative abundance of CBM tended to increase by 10.3% from the first to the second sampling (*p* = 0.063). A total of 347 CAZy level 2 genes were abundant at >0.001%, among which the GH with 136 GH genes dominated. Among the top functional subcategories at CAZy level 2 (Figure 4A), ANOVA showed that, at group level, the GMB comprised higher (*p* < 0.05) relative gene abundances of GT4, CMB50, CBM48, GH23, CE8, GH10, GT1, GT35, GH77 and GT28 at the second compared to the first fecal sampling at group level. At group level, the GMB also tended (*p* < 0.10) to be richer in GT2 and CBM13 but lower in GH2 and GH35 at the second sampling compared to 12 months earlier. Like for the taxonomic composition, differences in the shifts in functional gene abundances between samplings were detectable among the individual elephants (Figure 4A). For instance, at individual level, E4 had a 12.4% higher GMB50 family abundance compared to the other elephants at the first fecal sampling, whereas GH2 was 14.5% higher in E1 and E2 compared to the other three females at the second sampling.

**Figure 4:**
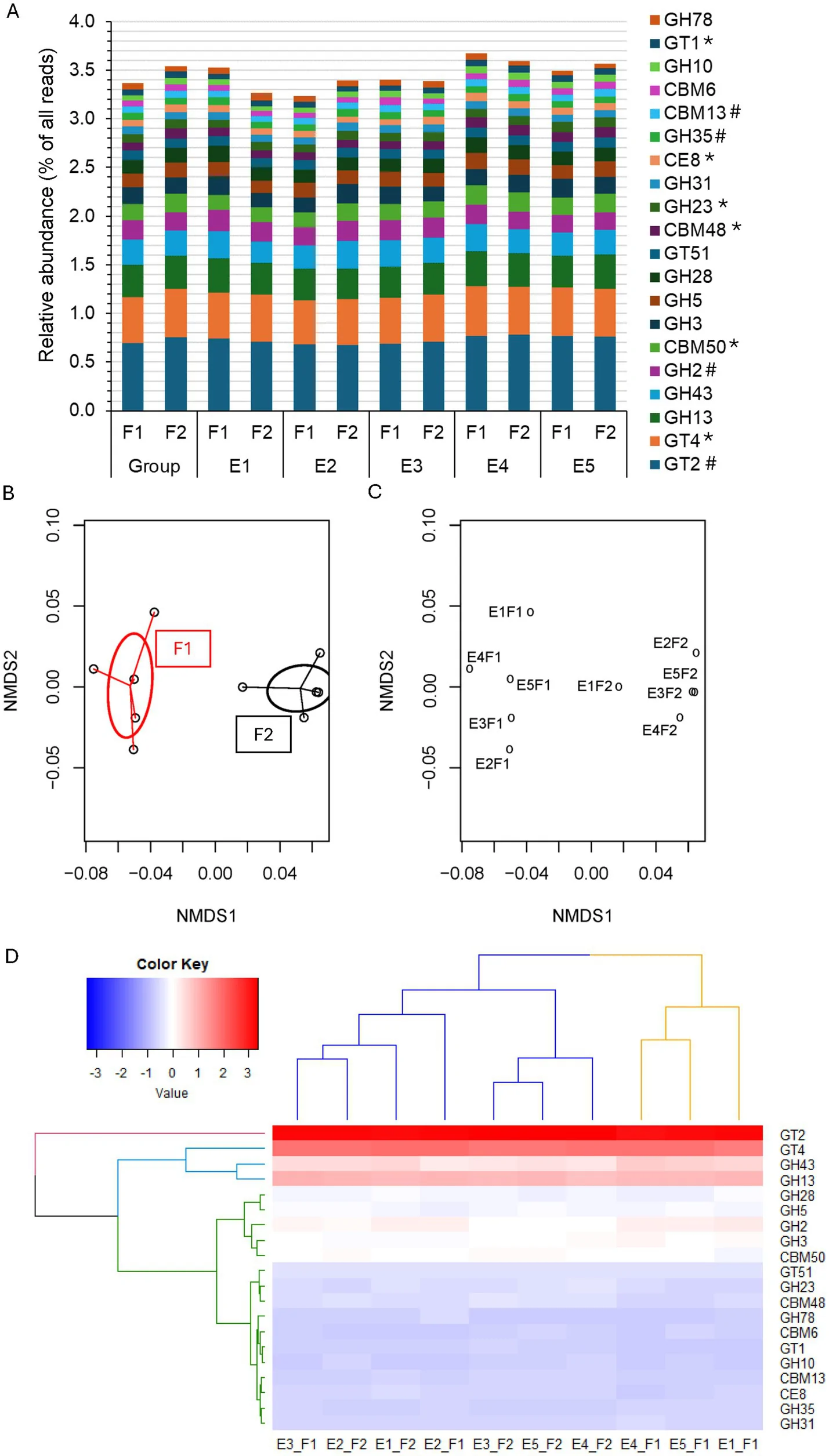
Comparison of the relative abundances of the 20 most abundant CAZy level 2 families at group and individual level between fecal collections **(A)**. F1, fecal collection in 2023; F2, fecal collection in 2024. E1, male; E2-E5, females. Group values for F1 and F2 are least-squares means; * *P* < 0.05; # 0.05 < *P* < 0.10 at group level. GT, glycosyl transferase; CBM, carbohydrate binding molecules; CE, carbohydrate esterase. Non-metric multidimensional scaling plots of fecal CAZy level 2 genes between fecal collections (**B**; stress = 0.0663) and fecal CAZy level 2 genes with assignment of the individual elephant **(C)**. Heatmap showing hierarchical clustering of CAZy level 2 genes across samples **(D)**. Relative abundance values were Z-score normalized and are represented on a color scale ranging from − 2 (blue, low abundance) to + 2 (red, high abundance). Rows represent CAZy level 2 genes. Columns represent fecal samples from individual elephants (E1-E5) and fecal collections (F1 and F2). Both CAZy level 2 genes and samples were clustered using hierarchical clustering to visualize similarities in composition.

The ANOVA further indicated at group level that alpha-diversity (Shannon) of CAZy level 2 genes was higher (*p* = 0.022) at the second sampling compared to 12 months earlier (Supplementary Table 8). The ANOSIM analysis at the CAZy level 2 suggested greater variation between sampling time points (*R* = 0.98, *p* < 0.01) than inter-animal variation (*R* = −0.45, *p* = 0.94), which was supported by beta-diversity. Beta-diversity analysis (Bray-Curtis distance) demonstrated distinguishable CAZy level 2 gene clusters between the two sampling time points (Figure 4B). Inter-animal shifts in CAZy level 2 and GH gene composition are presented in Figure 4C. Similar to the responses for taxonomy, hierarchical clustering showed differences between CAZy level 2 gene abundances between fecal collections and associations between individual elephants (Figure 4D). The comparison of relative abundances of the 20 most abundant GH gene families at group and individual level as well as results for the beta-diversity separated by sampling time point and assignment of the individual animal can be found in Supplementary Figure 1.

### 3.5 Integration of taxonomy, CAZymes and VFA

Identification of relationships between bacterial taxonomy with CAZy level 2 genes and VFA using horizontal sPLS-DA identified *Clostridium*, *Streptococcus* and *Coprobacillus* as discriminative genera for the first fecal sampling, whereas *Hallela* and *Prevotella* were the most discriminative genera for the second fecal sampling (Figure 5). For the CAZy level 2 genes, sPLS-DA identified CBM45, AA2 and AA5 as the most discriminative enzyme functions for the first sampling and CBM27 and CBM41 for the second fecal sampling. All VFA discriminated for the first fecal sampling, with butyrate being the most discriminative VFA. It associated positively with more CAZy level 2 families (i.e., CBM43, AA5, AA2, CBM18, CBM48, AA9, CBM45 and CBM50) than the other VFA. The VFA were negatively associated with seven genera, including *Hallela*, *Prevotella*, *Xylanibacter*, *Hoylesella*, *Palleniella*, *Segatella* and *Leyella*. These taxa were positively associated with each other but negatively with the discriminant functional genes.

**Figure 5:**
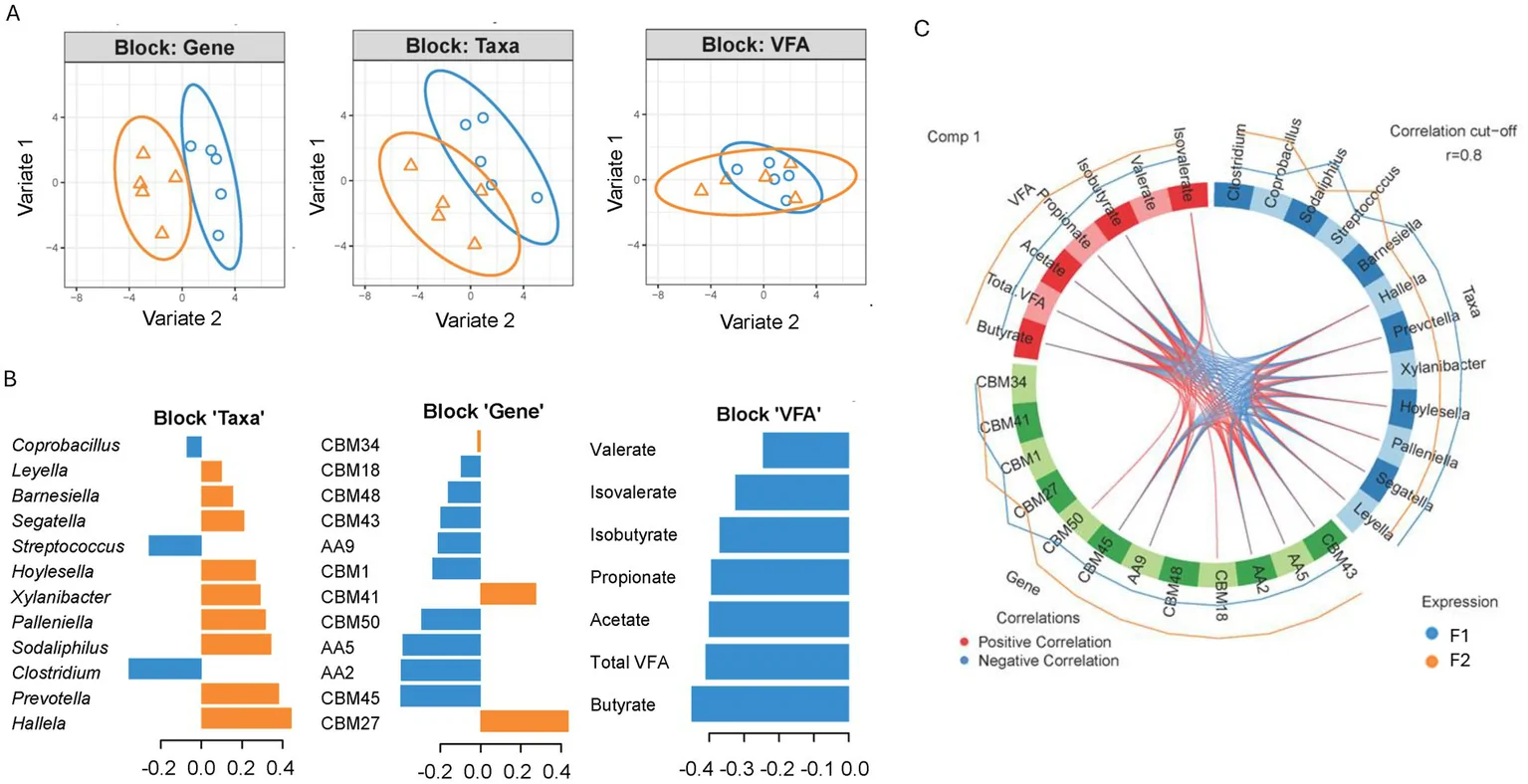
Identification and association of discriminant genera (>0.01% relative abundance), CAZy level 2 families (>0.001% relative abundance), and volatile fatty acids (VFA) using sparse partial least squares-discriminant analysis (sPLS-DA). **(A)** Sample plot, **(B)** Loading plot, and **(C)** Circos plot showing strongest associations between features for component 1 (|*r*| > 0.8). CBM, carbohydrate binding molecules; AA, auxiliary activities.

Integrating bacterial genera, GH families and VFA showed that *Lactobacillus*, GH23 and butyrate were the most discriminate features for the first fecal sampling (Supplementary Figure 2). The most discriminant features for the second fecal sampling were *Marseilla* and GH53. Butyrate was positively associated with GH23 but again only negatively with the identified discriminant taxa. *Prevotella* and *Xylanibacter*, as major fibrolytic genera, were positively associated with GH140, GH53, GH101, GH171 and GH173.

## 4 Discussion

In the present pilot study, we characterized the taxonomic and CAZy gene diversity and community structure of the GMB as well as fecal VFA in the small group of five adult captive African elephants at two occasions separated by 12 months apart. We determined the GMB in feces, which probably reflected more the community in the distal and less in the proximal sections of the hindgut. This, together with the small sample size, should be considered when interpreting the data. Overall, we anticipated differences in the GMB composition at taxonomic and functional level in the small but diverse elephant group from the first to the second sampling, which were probably due to changing environmental conditions between samplings (e.g., season, weather, diet and number of visitors at the zoo before sampling). Especially changes in food intake before fecal collection can be considered a major driver for variation in the GMB (7). However, we cannot exclude that other environmental factors also played a role in the present findings. To account for a potential effect of season in the present pilot study, we collected the samples in December in both years. Beta-diversity analysis supported that taxonomic shifts in the GMB community from the first to the second sampling were individual among elephants. At the functional level, in turn, shifts in the CAZy families seemed to be more similarly directed among elephants, suggesting that similar functional capacities in the GMB were associated with different taxonomic compositions. In this respect, although we did not quantify the actual intake of carrots by each individual elephant, the functional gene abundances reflected the higher amount of carrots in elephants’ diet before the second sampling as CAZy families related to the breakdown of carrot pectin were more abundant at the second compared to the first sampling.

Inter-animal differences in GMB composition have been reported in studies with wild and captive African elephants, caused by individual food choices, inter-animal competition and age (4, 17). In the present study, the external factors determining potential individuality were reduced at the zoo as elephants lived in the same ‘habitat’ and, on a daily basis, had access to the same foods. Nevertheless, individuals were able to select or reject offered foods, which may explain some individuality in the GMB composition. Moreover, the elephants differed in their origin, age and rank. The GMB may have become more closely matched through prolonged cohabitation of the five elephants, but it still seemed to be influenced by relationship, provenance and sex. This assumption was based on the greater similarities in the GMB communities (beta-diversity) between the matriarch (E4) and her daughters (E3 and E5), who maintained a close relationship. The GMB of the bull and the lower-ranking female (E2) clustered further apart in the NMDS plot from the three related females.

In contrast to the seasonal availability of food in the wilderness (17, 32), the zoo elephants were fed a more consistent and less varied diet based on fibrous hay and tree branches like in other zoological facilities and reserves (5, 17), which probably favored a more specialized GMB composition and corresponding metabolic features compared to their wild counterparts (3, 4, 8). In the wild, the diet of African elephants is less diverse during the wet season when vegetation ‘greens-up’, mainly based on grasses, whereas in the dry season the diet is broadened (23). The offered reed hay and branches were rich in lignocellulose. Vegetables and fruits, which provide more readily fermentable water-soluble carbohydrates and fiber, were mainly provided as ‘training rewards’ and therefore made up only a very small part of the zoo elephants’ diet at the first fecal sampling, but carrots became an integral part of elephants’ diet before the second sampling. Although we did not determine the individual intake of the various food items in the present pilot study, a certain competition due to hierarchy and individual preferences may have influenced the actual food intake of the zoo elephants.

While the focus in elephant GMB studies has been mostly on compositional changes, the dynamics in the total microbial load is an important component in the GMB-host interaction (33). The GMB of the five elephants showed a relatively stable number of bacterial gene copies across elephants and sampling time points. However, there were inter-individual as well as (over time) intra-individual differences in the gene copies of archaea, fungi and protozoa. These are key players in the breakdown of lignocellulose (34), and the higher amount of carrots in the diet seemed to have disadvantaged the fungi as indicated by the results at group level. When compared at individual level, their numbers were particularly lower in the female elephants. Carrot sugar and pectin fermentation, in turn, may have provided growth advantages for protozoa (35) and hydrogen-utilizing archaea (36) as indicated by the ANOVA at group level.

From a compositional point of view, bacteria and archaea dominated the GMB of the zoo elephants, whereas fungi, protozoa and viruses were in low abundance. Given the diet high in fiber, it is not surprising that the GMB was dominated by fibrolytic genera, such as *Prevotella*, *Bacteroides*, *Ruminococcus* and *Fibrobacter*, capable of breaking down complex plant polysaccharides including lignocellulose (37–39), which corroborates reports on the core GMB of other captive *Loxodonta* (5). Further (hemi-)cellulolytic genera (e.g., *Xylanibacter*, *Parabacteroides*, *Paraprevotella*, *Roseburia* and *Eubacterium*) (37) were among the 20 most abundant genera. Similar taxa but in different proportions have been reported for the core GMB of wild elephants (8, 40), which may be related to their diet and environment but also influenced by sample collection, DNA extraction method and sequencing technology used. For instance, hemicellulolytic genera such as *Oscillospiraceae* UCG-005 and *Rikenellaceae* dominated the GMB in wild elephants in Etosha National Park (40); genera that occurred in low abundance in the GMB of the five zoo elephants in the present pilot study.

In our study, *Treponema* was high abundant in the GMB of the zoo elephants, which was in contrast to previous reports on the composition of the core GMB in both captive and wild African elephants (4, 5, 11, 40). This spirochaete has been associated with cellulose degradation in the bovine rumen (41, 42). However, not all *Treponema* species contribute equally to the breakdown of plant polysaccharides (41–44). Taxonomy assignment at species level showed several uncultured *Treponema* species in the present study (data not shown), which renders it difficult to provide an accurate assessment about their contribution to fiber degradation. For instance, in co-culture *in vitro Treponema bryantii* attaches to fiber but it is *Fibrobacter succinogenes* which is the actual cellulolytic organism in the co-culture (42). These *Treponema* co-cultures appear to be more efficient regarding dry matter disappearance and VFA production than pure cultures of *F. succinogenes* (42). If these relationships are also true for the hindgut of elephants, similar co-associations may have occurred as both species were present in the elephant GMB (data not shown). Based on this assumption, it can be speculated that the high abundance of *Treponema* in the present GMB may have provided these elephants with an advantage in terms of maximizing their energy gain from cellulose-rich food. Other *Treponema* have been reported to have fructanolytic capacities and may have together with other taxa (e.g., *Bacteroides*, *Butyrivibrio* and *Pseudobutyrivibrio*) contributed to the breakdown of fructans in the meadow hay (44–47).

The higher proportion of carrots in the elephants’ diet before the second fecal sampling provided more water-soluble carbohydrates and pectins (e.g., α-1,4-linked galacturonans and rhamnogalacturonans) (48) to the GMB. Visual examination of the feces showed carrot pieces and confirmed their consumption by all elephants. According to the performed sPLS-DA and the identified discriminative taxa, the readily fermentable carbohydrates may have provided *Clostridium*, *Coprobacillus*, *Streptococcus* and *Lactobacillus* a direct or indirect (through cross-feeding) growth advantage, as they were more abundant in the GMB at the second sampling compared to the first fecal sampling as indicated by ANOVA (group level) and sPLS-DA. In contrast and also indicated by ANOVA and sPLS-DA, taxa capable of breaking down more complex plant polysaccharides including cellulose and arabinoxylans, such as *Bacteroides* and several genera within *Prevotellaceae* (i.e., *Hallella*, *Prevotella*, *Xylanibacter*, *Palleniella*, *Hoylesella*, *Segatella* and *Leyella*) (49) were less abundant in the GMB at the second fecal samples, which may have been related to a higher consumption of carrots.

As elephants derive their nutrition exclusively from plant material, their GMB encodes unique CAZy genes to efficiently hydrolyze a variety of plant-derived polymers (50, 51). The importance of GH for plant biomass breakdown was highlighted by the number of GH families present and their predominance in the GMB, which supports previous findings for captive African elephants (5, 17). Among the 20 most abundant GH families, GH43, GH2 and GH28 families comprised hemicellulolytic and pectinolytic activities, and GH3, GH5, GH10, GH51 and GH16 families included cellulolytic activities (50–52). While the ANOVA at group level showed no difference in the alpha-diversity at taxonomic level from the first to second sampling, there was a diversification at CAZy level 2. Beta-diversity further supported a different CAZy level 2 composition in the second compared to the first year, which speculatively may be due to the dietary changes from the first to the second sampling. In assuming that the major effect on the GMB was the diet, the ANOVA at CAZy level 1 (group level) alluded that the effect of the dietary change seemed to be greater on relative abundance of GT gene families than on GH gene families. Accordingly, the ANOVA at CAZy level 2 demonstrated at group level that the higher amount of carrots in elephants’ diet tended to increase or increased the most abundant GT families (GT2 and GT4), which are transferases for glycosyl, fucosyl and rhamnose alpha-1,3-glycosyl residues (52). Regarding the GH families, *Clostridium* and *Streptococcus* encode genes belonging to the GH77 family (glucanotransferases) (52), potentially explaining their higher abundance at the second sampling. This finding together with the CBM and AA families identified as discriminant by the sPLS-DA for the second sampling were indicative of the breakdown of hemicelluloses. Likewise, the sPLS-DA performed on the GH families as functional component identified GH families for breakdown of carrot pectin, specifically rhamnogalacturonan (i.e., GH78 and GH140), and families for the breakdown of arabinogalactans (i.e., GH53 and GH147), which may have originated from the tree branches. The strong positive association of certain discriminant GH families (e.g., GH53 and GH78) with hemicellulolytic genera within *Prevotellaceae* may point to the origin of the functional characteristics, which should be followed up in future studies.

Despite being a useful proxy for host energy provision (53, 54), fecal VFA have been rarely studied in connection with the GMB in elephants. The present concentrations of about 70 to 100 mmol/kg feces were in the range or higher than in ruminants (55, 56) but lower than in captive rhinoceros (57). Aside from the VFA production itself, gut length, retention time and absorptive capacity are important intrinsic factors for the energy supply to the host (15, 16); factors that we cannot completely disentangle by means of the current data. In contrast to the CAZy results and against our expectation, the higher amount of carrots in elephants’ diet did not raise fecal VFA concentrations at group level. Comparing fecal VFA at individual level may provide some clues for the missing dietary effect at group level as the dietary effect was opposite among elephants. The matriarch (E4) and her two daughters (E3 and E5) had higher VFA concentrations in their feces. By contrast, female E2 (lower ranking female) had a lower VFA concentration in feces at the second sampling. If concentration in feces and intestinal absorption correlate as in other hindgut fermenting species (58), it can be assumed that the GMB of the related females had a higher fermentative capacity to extract energy from the consumed foods at the second sampling. In line with this assumption is the high similarity in the CAZy level 2 gene composition (but not in taxonomy) of the three related females as indicated by their close proximity in the NMDS plots. Due to the short gut transit time in African elephants (15), it can be speculated whether the VFA concentrations may also reflect the food intake in the past 20 to 30 h. Since the elephants were housed together, there is a certain probability that the matriarch, accompanied by her daughters, ate more before the lower-ranking female E2 was allowed to eat her share of food, but as we did not quantify the food intake this remains speculative. The strong increase in its concentration in feces of the three related females may be behind the fact that the sPLS-DA identified butyrate as most discriminant VFA between sampling time points. Among the straight-chained VFA, butyrate is the most beneficial VFA for the metabolism of enterocytes, triggering anti-inflammatory signal transduction pathways and acting as primary energy source (59). There were only negative associations between VFA and the discriminant taxa. Given that the discriminating taxa and functional features were related to cellulose and arabinoxylan breakdown, these could be representative of the lower fermentability of the substrate, which may explain the negative association with the VFA.

## 5 Conclusion

As expected, bacterial genera involved in plant polysaccharide breakdown including cellulose and corresponding CAZy genes dominated the GMB of the five captive elephants, reflecting their high-fiber diet. The high abundance of *Treponema* in the present GMB may represent an adaptation to the high-fiber diet and may have provided the elephants an advantage in obtaining energy from lignocellulose. Shifts in CAZy gene families were more similarly directed than taxonomic shifts in the GMB from the first to the second sampling, indicating individual GMB composition but similar functional potential among elephants. Moreover, the present results on inter-animal variation in GMB composition emphasize the importance of individual animal-based solutions (e.g., feeding) to promote health and welfare of captive elephants, and may lay the ground for hypotheses formulation and testing in wild elephants.

## Data Availability

The original contributions presented in the study are publicly available. This data can be found in the NCBI repository under accession number: PRJNA1452568.
