# Supplementary material for: Fibrolytic capacity of the gut microbiome in captive African elephants (Loxodonta africana): a pilot study
Source: Front Vet Sci. 2026 Sep 30;13:1933152. doi: 10.3389/fvets.2026.1933152 (PMC13663176; doi:10.3389/fvets.2026.1933152)
Supplement: Supplementary file 1 [file Table_1.docx]

Supplementary Material

**Supplementary Table 1.** Elephants in Vienna Zoo.

| Elephant | Born | Sex | in Vienna Zoo since | Relationship |
| --- | --- | --- | --- | --- |
| E1 | 2001 | Male | 2023 | Unrelated |
| E2 | 1992 | Female | 2009 | Unrelated |
| E3 | 2013 | Female | born in Vienna Zoo | Daughter of E4 |
| E4 | 1985 | Female | 1998 | Matriach |
| E5 | 2003 | Female | born in Vienna Zoo | Daughter of E4 |

**Supplementary Table 2**. Oligonucleotide primers used for microbial quantification.

| Primer |  | Sequence 5’-3’ | Product size (bp) | Eff. (%) | Reference |
| --- | --- | --- | --- | --- | --- |
| Universal bacteria (16S rRNA gene) | 341-357F | CCTACGGGAGGCAGCAG | 189 | 99 | Muyzer et al., 1993 |
|  | 518-534R | ATTACCGCGGCTGCTGG |  |  |  |
| Total protozoa (18S rRNA gene) | 316f | GCTTTCGWTGGTAGTGTATT | 233 | 99 | Sylvester et al., 2004 |
|  | 539r | CTTGCCCTCYAATCGTWCT |  |  |  |
| Total methanogens (16S rRNA gene) | uniMet1-F | CCGGAGATGGAACCTGAGAC | 160 | 103 | Zhou et al., 2009 |
|  | uniMet1-R | CGGTCTTGCCCAGCTCTTATTC |  |  |  |
| Universal fungi (D1 domain of 26S rRNA gene) | NL1 | GCA TAT CAA TAA GCG GAGGAAAAG | 250 | 97 | Urubschurov et al., 2015 |
|  | LS2 | ATTCCCAAACAACTCGACTC |  |  |  |

Eff., efficiency.

**References**

Muyzer, G., De Waal, E. C. & Uitterlinden, A. G. Profiling of complex microbial populations by denaturing gradient gel electrophoresis analysis of polymerase chain reaction-amplified genes coding for 16S rRNA. *Appl. Environ. Microbiol.* 59, 695–700 (1993).

Sylvester, J. T., Karnati, S. K. R., Yu, Z., Morrison, M. & Firkins, J. L. Development of an assay to quantify rumen ciliate protozoal biomass in cows using real-time PCR. *J. Nutr*. 134, 3378–3384 (2004).

Zhou, M., Hernandez-Sanabria, E. & Le, L. G. Assessment of the microbial ecology of ruminal methanogens in cattle with different feed efficiencies. Appl. Environ. Microbiol. 75, 6524–6533 (2009).

Urubschurov, V., Büsing, K., Janczyk, P., Souffrant, W. B. & Zeyner, A. Development and evaluation of qPCR assay for quantitation of Kazachstania slooffiae and total yeasts occurring in the porcine gut. Curr. Microbiol. 71, 373–381 (2015).

**Supplementary Table 3.** Oven temperature program for the volatile fatty analysis on the gas chromatograph.

| Retention time in min. | rate in °C/min. | Target in °C | Hold in min. |
| --- | --- | --- | --- |
| 0 | 0 | 90 | 0 |
| 11 | 10 | 200 | 0 |
| 12.16 | 125 | 220 | 0 |
| 12.2 | Stop |  |  |

**Supplementary Table 4.** Limits of detection and quantification of volatile fatty acids with and without matrix (intestinal digesta).

|  | µMol/mL | | µMol/mL | |
| --- | --- | --- | --- | --- |
|  | LOD without Matrix | LOQ without Matrix | LOD mit Matrix | LOQ mit Matrix |
| Acetic acid | 0.0182 | 0.164 | 0.091 | 0.821 |
| Propionic acid | 0.0021 | 0.018 | 0.010 | 0.092 |
| i-Butyric acid | 0.0012 | 0.010 | 0.006 | 0.052 |
| n-Butyric acid | 0.0013 | 0.011 | 0.006 | 0.057 |
| i-Valeric acid | 0.0008 | 0.007 | 0.004 | 0.034 |
| n-Valeric acid | 0.0012 | 0.011 | 0.006 | 0.054 |
| Caproic acid | 0.0011 | 0.010 | 0.006 | 0.050 |
| Heptanoic acid | 0.0023 | 0.021 | 0.011 | 0.103 |
| 4-methylvaleric acid (internal standard) | 0.0012 | 0.010 | 0.006 | 0.052 |

**Supplementary Table 5.** Short-chain fatty acid (SCFA) concentrations in feces in 2023 (F1) and 2024 (F2) at group level.

| Concentration (mmol/kg) | F1 | F2 | SEM | *P*-value |
| --- | --- | --- | --- | --- |
| Total SCFA | 76.7 | 82.6 | 5.10 | 0.341 |
| Acetate | 53.1 | 57.0 | 3.42 | 0.356 |
| Propionate | 17.1 | 18.5 | 1.32 | 0.323 |
| Isobutyrate | 0.62 | 0.65 | 0.05 | 0.504 |
| Butyrate | 4.76 | 5.21 | 0.34 | 0.289 |
| Isovalerate | 0.65 | 0.68 | 0.06 | 0.550 |
| Valerate | 0.52 | 0.55 | 0.05 | 0.486 |
| Caproate | 0 | 0 | - | - |
| Heptanoate | 0 | 0 | - | - |

Values are least-squares means ± standard error of the mean (SEM).

**Supplementary Table 6.** Molar proportions of short-chain fatty acids in feces of individual elephants (E1, male; E2-E5, females) collected in December 2023 (F1) and 2024 (F2).

|  | E1 | | E2 | | E3 | | E4 | | E5 | |
| --- | --- | --- | --- | --- | --- | --- | --- | --- | --- | --- |
| % of total VFA | F1 | F2 | F1 | F2 | F1 | F2 | F1 | F2 | F1 | F2 |
| Acetate | 69.5 | 69.4 | 68.5 | 68.9 | 70.3 | 68.6 | 67.5 | 67.6 | 70.5 | 70.6 |
| Propionate | 22.4 | 22.6 | 22.6 | 22.3 | 21.2 | 22.3 | 23.6 | 23.7 | 21.2 | 21.1 |
| Isobutyrate | 0.66 | 0.67 | 0.87 | 0.88 | 0.84 | 0.84 | 0.86 | 0.75 | 0.81 | 0.82 |
| Butyrate | 6.12 | 6.11 | 6.23 | 6.14 | 6.25 | 6.79 | 6.31 | 6.39 | 6.11 | 6.09 |
| Isovalerate | 0.65 | 0.65 | 1 | 1 | 0.84 | 0.87 | 0.94 | 0.81 | 0.81 | 0.83 |
| Valerate | 0.61 | 0.59 | 0.86 | 0.86 | 0.59 | 0.62 | 0.75 | 0.69 | 0.6 | 0.61 |
| Caproate | 0 | 0 | 0 | 0 | 0 | 0 | 0 | 0 | 0 | 0 |
| Heptanoate | 0 | 0 | 0 | 0 | 0 | 0 | 0 | 0 | 0 | 0 |

.

**Supplementary Table 7.** Relative abundance (%) of 30 most abundant phyla in feces across elephants and fecal collections.

| Taxon | Average relative abundance  (% of all reads) |
| --- | --- |
| k__Bacteria;p__Bacteroidota | 29.51 |
| k__Bacteria;p__Bacillota | 22.23 |
| k__Bacteria;p__Spirochaetota | 5.40 |
| k__Bacteria;p__Lentisphaerota | 3.00 |
| k__Viruses;p__Uroviricota | 2.53 |
| k__Bacteria;p__Verrucomicrobiota | 1.97 |
| k__Bacteria;p__Fibrobacterota | 1.89 |
| k__Archaea;p__Euryarchaeota | 1.38 |
| k__Bacteria;p__Mycoplasmatota | 0.98 |
| k__Bacteria;p__Pseudomonadota | 0.78 |
| k__Bacteria;p__Actinomycetota | 0.55 |
| k__Bacteria;p__Planctomycetota | 0.46 |
| k__Bacteria;p__Thermodesulfobacteriota | 0.23 |
| k__Bacteria;p__Synergistota | 0.21 |
| k__Archaea;p__Candidatus Thermoplasmatota | 0.27 |
| k__Bacteria;p__Candidatus Saccharibacteria | 0.19 |
| k__Bacteria;p__Candidatus Melainabacteria | 0.13 |
| k__Bacteria;p__Kiritimatiellota | 0.09 |
| k__Bacteria;p__Chloroflexota | 0.08 |
| k__Bacteria;p__Myxococcota | 0.06 |
| k__Bacteria;p__Campylobacterota | 0.07 |
| k__Eukaryota;p__Chytridiomycota | 0.07 |
| k__Bacteria;p__Acidobacteriota | 0.05 |
| k__Bacteria;p__Cyanobacteriota | 0.05 |
| k__Bacteria;p__Fusobacteriota | 0.05 |
| k__Bacteria;p__Armatimonadota | 0.03 |
| k__Bacteria;p__Thermotogota | 0.02 |
| k__Bacteria;p__Elusimicrobiota | 0.02 |
| k__Bacteria;p__Ignavibacteriota | 0.02 |
| k__Bacteria;p__Nitrospirota | 0.02 |
| k__Viruses;p__Phixviricota | 0.01 |
| k__Bacteria;p__Chlorobiota | 0.01 |
| k__Bacteria;p__Chlamydiota | 0.01 |
| k__Bacteria;p__Candidatus Hydrogenedentota | 0.01 |
| k__Bacteria;p__Candidatus Cloacimonadota | 0.01 |

**Supplementary Table 8.** Comparison of alpha-diversity indices (Shannon and Simpson) for microbial genera and carbohydrate-active enzyme (CAZy) level 2 families in elephant feces collected in 2023 (F1) and 2024 (F1) at group level.

| Item | F1 | F2 | SEM | P-value |
| --- | --- | --- | --- | --- |
| Genera |  |  |  |  |
| Shannon | 3.82 | 3.84 | 0.042 | 0.867 |
| Simpson | 0.94 | 0.95 | 0.004 | 0.581 |
| CAZy level 2 |  |  |  |  |
| Shannon | 4.31 | 4.36 | 0.008 | 0.022 |
| Simpson | 0.97 | 0.97 | 0.001 | 0.225 |

Values are least-squares means ± standard error of the mean (SEM).

**Supplementary Table 9.** Comparison of relative abundance of carbohydrate-active enzymes (CAZy) level 1 in feces collected in 2023 (F1) and 2024 (F2) at group level.

| CAZy level 1 (% of all functional genes) | 2023 | 2024 | SEM | P-value |
| --- | --- | --- | --- | --- |
| Glycoside hydrolases | 2.91 | 2.96 | 0.051 | 0.545 |
| Glycosyl transferases | 1.87 | 2.25 | 0.031 | 0.002 |
| Carbohydrate binding modules | 0.66 | 0.73 | 0.017 | 0.063 |
| Carbohydrate esterases | 0.32 | 0.33 | 0.010 | 0.339 |
| Polysaccharide lyases | 0.11 | 0.11 | 0.010 | 0.571 |
| Auxiliary activities | 0.02 | 0.04 | 0.002 | 0.001 |

Values are least-squares means ± standard error of the mean (SEM).

**Supplementary Figure 1.** Comparison of the relative abundances of the relative abundances of the 20 most abundant glycoside hydrolases (GH) at group and individual level between fecal collections (A). F1, fecal collection in 2023; F2, fecal collection in 2024. E1, male; E2-E5, females. * *P* < 0.05; # 0.05 < *P* < 0.10 at group level. Non-metric multidimensional scaling plots of GH composition in elephant feces between fecal collections (B; stress = 0.0531), and with assignment of the individual elephant (C).

**Supplementary Figure 2.** Identification and association of discriminant genera (>0.01% relative abundance), glycoside hydrolase (GH) families and volatile fatty acids (VFA) using sparse partial least squares-discriminant analysis (sPLS-DA). A) Sample plot, B) loading plot, and C) circos plot showing strongest associations between features for component 1 (|r| > 0.8).
